# Performance of High-Quality Point-of-Care Ultrasound by Advanced Practice Providers in an Academic Emergency Department

**DOI:** 10.7759/cureus.81564

**Published:** 2025-04-01

**Authors:** Jamie Aranda, Robert Treat, Daniel Dwyer, Jennifer Trimboli, Mary Beth Phelan

**Affiliations:** 1 Emergency Medicine, Medical College of Wisconsin, Milwaukee, USA

**Keywords:** advanced practice providers, emergency medicine, image quality, nurse practitioner, physician assistant, point-of-care ultrasound, ultrasound training

## Abstract

Introduction

Point-of-care ultrasound (POCUS) is a valuable tool in emergency medicine, enhancing diagnostic accuracy and guiding patient care. While emergency physicians have long utilized POCUS, advanced practice providers (APPs), including physician assistants and nurse practitioners, were only formally supported by the American College of Emergency Physicians in 2019. This study evaluates the impact of an educational intervention on APPs' ability to perform high-quality, billable POCUS in a high-volume academic emergency department (ED) (75,000 visits/year).

Materials and methods

This study included three components: (1) a one-hour didactic session followed by hands-on training and direct clinical observation in soft tissue (ST) and fetal heart tone (FHT) POCUS, (2) a quality assurance review of scans performed by APPs, and (3) a voluntary survey assessing APP's perceptions of POCUS use. Data were collected between 2015 and 2018. Descriptive statistics were used to analyze the survey data. Survey responses were reported as frequencies. Descriptive statistics, including percentages, means for interval scales, and medians for ordinal scales, were generated using SPSS Statistics version 26.0 (IBM Corp. Released 2019. IBM SPSS Statistics for Windows, Version 26.0. Armonk, NY: IBM Corp).

Results

Fourteen APPs performed 471 POCUS exams, with 87% (N=412) initially meeting criteria for billing. The remaining 13% (N=59) scans required additional steps, either completion of electronic health record documentation in 87% (N=51) or an attending physician signature in 13% (N=8), before they were considered billable. Following standard departmental procedures for addressing charting deficiencies, all required documentation and signatures were completed, making 100% of scans ultimately billable, with no rejections due to image quality. Survey respondents (N=6) reported increased confidence and changes in clinical management due to POCUS. The primary barrier to POCUS use was limited training in additional applications. APPs used POCUS more frequently for ST evaluations than for FHT assessments, with self-reported confidence scores of 7.8 and 4.4 out of 10, respectively.

Conclusion

This study demonstrates that APPs can perform high-quality, billable POCUS in the ED following structured training and quality review feedback.

## Introduction

Point-of-care ultrasound (POCUS) is a critical diagnostic tool widely used by emergency physicians to enhance diagnostic accuracy and guide patient care [1]. While emergency physicians have long utilized POCUS, its training, practice, and integration by advanced practice providers (APPs), including physician assistants and nurse practitioners, was only formally supported by the American College of Emergency Physicians in 2019. [2]. However, APPs, including physician assistants (PAs) and nurse practitioners (NPs), are not required to undergo standardized residency or fellowship training to practice in EM, resulting in variable POCUS experience and proficiency. Most APPs receive on-the-job training to acquire POCUS skills [3,4]. Given the expanding use of POCUS in emergency care [1] paralleling increased integration of APPs into the emergency department (ED) [5-8], there is substantial opportunity for APPs to incorporate POCUS into their clinical practice.

Despite this potential, formal POCUS training for APPs remains limited. While studies suggest that APPs can accurately obtain and interpret ultrasound images with appropriate training [1,9,10-13], the lack of standardized education programs is a persistent barrier to POCUS use [3,4,8,13-15]. PAs are required to complete ED rotations. However, no such requirement exists for NPs [16,17]. Furthermore, only 23% of Accreditation Review Commission on Education for the Physician Assistant programs integrate POCUS training into their curricula, with no equivalent mandate for NP programs [3]. Specialized certifications, such as the PA Certificate of Added Qualifications in EM, include limited POCUS requirements, whereas post-graduate NP programs specify general POCUS knowledge but lack detailed modality guidelines [18,19]. In response to this gap, some institutions develop on-the-job training and credentialing programs for APP POCUS use, aligning with the American College of Emergency Physicians policy supporting APP training and integration into emergency ultrasound programs [10,17]. Our institution's APPs historically relied on EM physicians to perform POCUS exams, creating a bottleneck in the "minor care/fast track" area where APPs work independently. The most frequently utilized POCUS modalities in this setting were soft tissue (ST) and limited abdominal ultrasound in pregnancy for fetal heart tone (FHT) assessment. To address this gap, we sought to evaluate whether APPs could perform clinically indicated, high-quality, billable ST and FHT POCUS and to explore the reported benefits, barriers, and confidence in their use of ultrasound.

## Materials and methods

This mixed-methods study included a simulation-based POCUS training session for APPs, a retrospective review of APP-performed ST and FHT POCUS for quality assurance (QA) and billing purposes, and a voluntary survey completed by APPs. The study was conducted at the Medical College of Wisconsin, Froedtert Hospital Department of Emergency Medicine, Milwaukee, Wisconsin, United States. This site is a large Midwestern academic ED, with an annual volume of 75,000 visits at the time of the study. The ED employs PAs and NPs who manage patients in low- and high-acuity areas. This protocol was approved by the Institutional Review Board of Medical College of Wisconsin, Froedtert Hospital, for human research (approval number: PRO00033570).

POCUS training

In 2014, all ED APPs were invited to participate in voluntary simulation-based training sessions led by faculty from the Division of Emergency Ultrasound. Two training sessions focused on applying ST and FHT POCUS, respectively, and consisted of a didactic lecture and hands-on practice. Additionally, a follow-up hands-on POCUS session was integrated into a scheduled APP meeting to support skill retention and continued competency development. Didactic instruction was provided by an ultrasound fellowship-trained emergency physician and covered normal and abnormal sonographic anatomy, incidental findings, and image interpretation. Following the didactic session, participants engaged in a one-hour practical session using the same ultrasound machine available in the ED. During this session, APPs practiced fundamental skills such as familiarizing themselves with the US machine functions, image acquisition and optimization (including adjusting gain and depth), and applying M-mode and color flow Doppler. Standardized patients and a low-fidelity simulator created by the instructor were used to facilitate hands-on practice and ensure proficiency in image acquisition.

To reinforce learning and assess competency, APPs were required to complete 10 supervised POCUS examinations (for each modality) on ED patients under direct supervision by ultrasound-credentialed, emergency physician faculty. Completing this supervised scanning process led to credentialing for independent performance of POCUS. Supervising EM faculty were required to co-sign all POCUS documentation. Our hospital requires the direct supervision of at least three procedures performed by the APP in order to attain category II privileges for most procedures. We felt that POCUS represented an advanced category II procedure and could not be adequately assessed after only three iterations. Therefore, we required a minimum completion of 10 successful scans for each modality. This decision was supported by the APP director and the Director of Emergency Ultrasound within the Department of EM. Upon completing this process, APPs were credentialed to perform ST and/or FHT POCUS independently. Supervising EM faculty were required to co-sign all POCUS documentation.

Quality assurance and image review

The ED's QA process involves reviewing all POCUS scans for image quality, diagnostic accuracy, documentation, and medical decision-making. Patient identification and image review utilized a pre-existing database created with Filemaker Pro Advanced (Claris International Inc., Sunnyvale, CA, USA), Change Healthcare Radiology Solutions Radiology Station Lite imaging software (Change Healthcare, Nashville, TN, USA), and the electronic health record (EHR) (Epic Systems, Verona, WI, USA). All scans performed by APPs in the ED were required to be clinically indicated and were reviewed by an ultrasound fellowship-trained, board-certified emergency physician. Indications for ST POCUS included evidence of ST infection, evidence of ST mass, or suspected foreign body, and the indication for FHT POCUS was the inability to obtain a fetal heartbeat using bedside doppler in a patient with a documented intrauterine pregnancy.

QA feedback on scan quality, diagnostic accuracy, and documentation was communicated directly to the APPs, supervising attending physicians, and EM billing staff via email. Data was collected from 2015 to 2018, tracking the total number of scans performed by APPs and the billable proportion. To qualify as billable, ST ultrasound scans needed to include two views (transverse and sagittal) of the target area, with Doppler imaging when appropriate. For FHT scans, billable criteria included two views of the target area and using M-mode to trace and calculate the fetal heart rate. Documentation requirements included a clear indication and a statement on the presence or absence of a fetal heartbeat. Supervising faculty were also required to co-sign the documentation. Using prebuilt smart phrases, documentation in the EHR was required to specify the indication and a final impression consistent with the clinical scenario and images. Faculty co-signature of the final documentation was mandatory.

Survey

A novel electronic survey (see supplemental materials) was created using Qualtrics (Qualtrics LLC, Provo, UT, USA). The survey addressed participants' current and past POCUS training, the impact of ultrasound on their clinical practice, and perceived benefits or barriers to POCUS use. It included a mix of free-text, Likert-type scale, and multiple-choice questions. Survey questions were developed by the study authors and reviewed for necessity, relevance, and clarity with a content expert in evaluating and measuring educational outcome data. APPs were invited to participate in an anonymous and voluntary survey comprising 15 questions. Informed consent was detailed in the invitation email containing the survey link. Consent was implied by survey completion. Three reminder emails were sent via the department email account to maximize participation.

Statistical analysis

Descriptive statistics were used to analyze the survey data. Survey responses were reported as frequencies. Descriptive statistics, including percentages, means for interval scales, and medians for ordinal scales, were generated using SPSS Statistics version 26.0 (IBM Corp. Released 2019. IBM SPSS Statistics for Windows, Version 26.0. Armonk, NY: IBM Corp).

## Results

Billable ultrasounds

**Figure 1:**
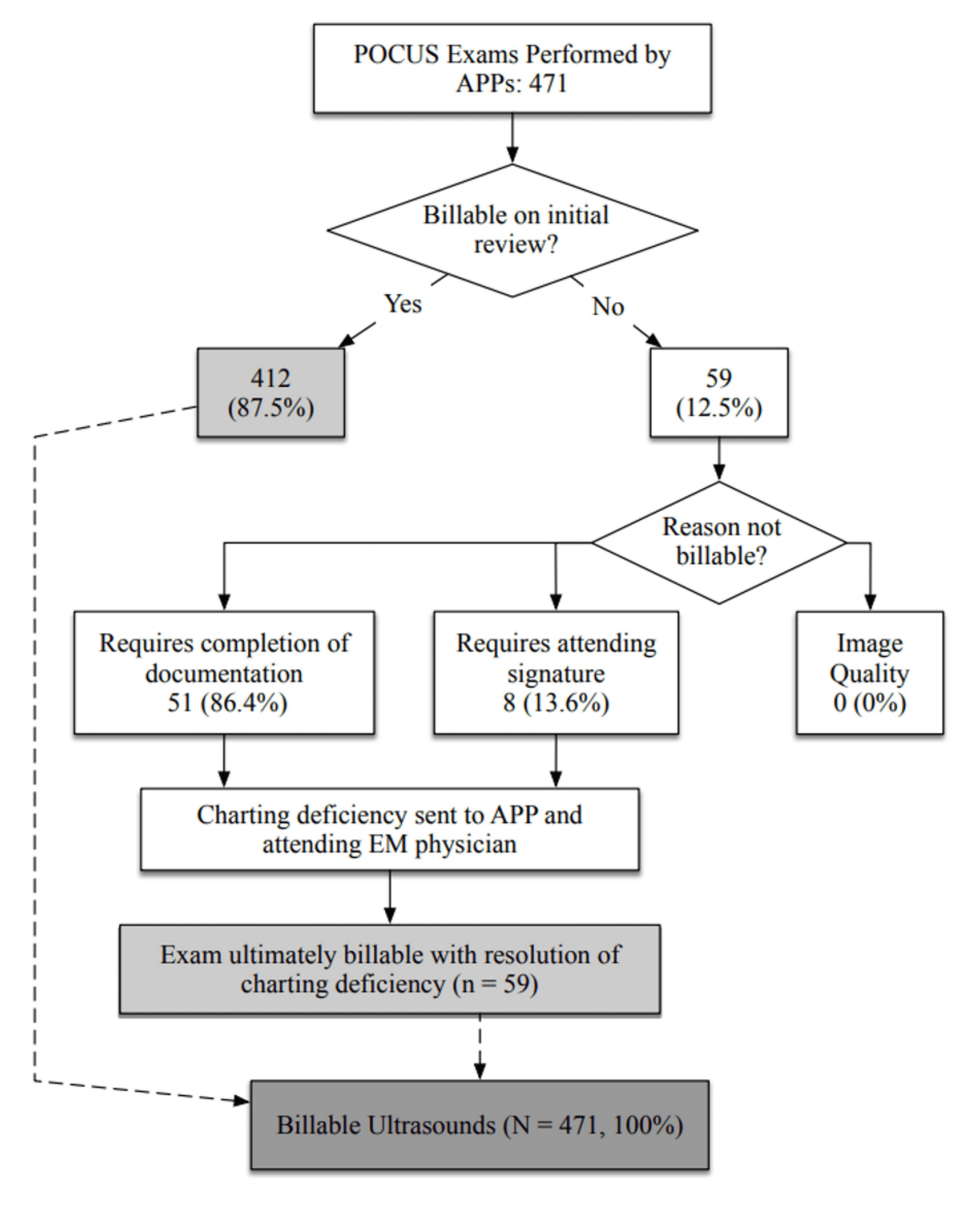
Billable status of POCUS performed by APPs in the ED between 2015 and 2018 POCUS: point-of-care ultrasound, APPs: advanced practice providers, ED: emergency department, EM: emergency medicine

Between 2015 and 2018, APPs performed a total of 471 POCUS examinations (Figure 1). Upon initial quality review, 412 scans (87%) met the criteria for immediate billing. The remaining 59 scans (48 ST, 8 FHT, and 3 FAST) required additional steps, either completion of EHR documentation or an attending physician's signature, before they were considered billable.

Of the 59 initially non-billable scans, 51 (87.5%) were due to missing APP documentation, an issue that occurred at least once for all APPs. The remaining eight scans were unbillable at the initial review due to the absence of a faculty physician's signature. Following standard departmental procedures for addressing charting deficiencies, all required documentation and signatures were completed, making 100% of scans ultimately billable. Importantly, no scans were deemed unbillable due to inadequate image quality. Fourteen APPs were employed by the department of EM and were invited to complete ST and FHT POCUS training (Table 1). Thirteen APPs were employed at the time the survey was administered.

**Table 1:** Training and experience POCUS: point-of-care ultrasound, FAST: focused assessment with sonography for trauma, OB: obstetrics, GI: gastrointestinal, LP: lumbar puncture, RUQUS: right upper quadrant ultrasound, EFAST: extended focused assessment with sonography for trauma, LOS: length of stay, PA: physician assistant, NP: nurse practitioner, MSK: musculoskeletal

Question	Answer (n=6)
What is your background training?	PA (5)
	NP (1)
List any specialty training you have in your field (such as fellowship or residency training).	Fellowship (1)
How many years have you been in practice? (including residency and fellowship training if applicable)	5 years (1)
	6 years (1)
	6.5 years (1)
	7.5 years (1)
	19 years (1)
	20 years (1)
	Average of all responses: 10.7 years (n=6)
How many years have you been using POCUS?	2 years (2)
	3 years (1)
	4 years (1)
	5 years (1)
	8 years (1)
	Average of all responses: 4 years (n=6)
Which additional applications of POCUS would you be interested in learning?	“Cardiac” (1)
	“Cardiac, FAST, ophthalmology” (1)
	“FAST, cardiac, and OB for fetal cardiac activity” (1)
	“Regional blocks, GI/appendix” (1)
	“Testicular, advanced procedure guidance such as central line, LP” (1)
	“Vascular” (1)
In which additional applications are you personally utilizing POCUS?	“Renal, RUQUS, MSK, aorta, EFAST, ocular” (1)
Where did you learn your POCUS skills? (select all that apply)	Fellowship (1)
	Paid ultrasound course (1)
	On-the-job training or training program (6)
Do you bill for your ultrasounds?	Yes (2)
If there are additional benefits to performing POCUS not listed, please include them here.	“Decreases LOS or time to disposition, patient education” (1)
	“Less complications (cutting into an “abscess" that has nothing to drain)” (1)

Survey results

**Figure 2:**
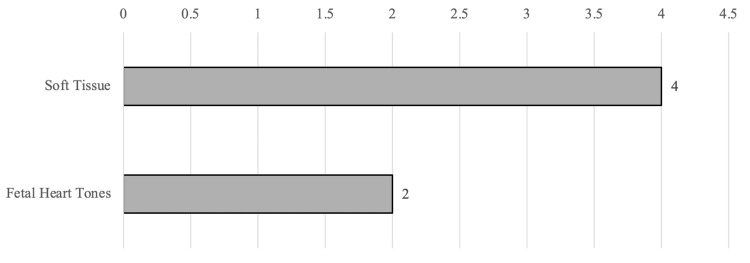
Median APP responses to the question, “How often do you personally use the POCUS application?” Responses are based on a 5-point scale: 1 = never, 2 = rarely, 3 = sometimes, 4 = often, and 5 = very often APP: advanced practice provider, POCUS: point-of-care ultrasound

The survey was completed by six out of 13 APPs (46%). Five PAs completed the survey, and one NP completed the survey. The mean years of experience was 10.7 years, and the mean years of experience performing ultrasound was 4.0 years. All APPs completing the survey reported learning POCUS in “on-the-job training or training programs offered through my work” (n=6, 100%). One reported completing a US fellowship, and one reported completing a formal paid ultrasound course. Only two APPs were aware that POCUS exams performed in the ED were billed (Table 1). Four APPs (66.7%) reported using ST POCUS “often” or “very often,” while only one reported using FHT POCUS “often” or “very often.” The median response for use of ST POCUS was 4 (very often), and the median response for use of FHT POCUS was 2 (rarely) (six responses), as seen in Figure 2.

**Figure 3:**
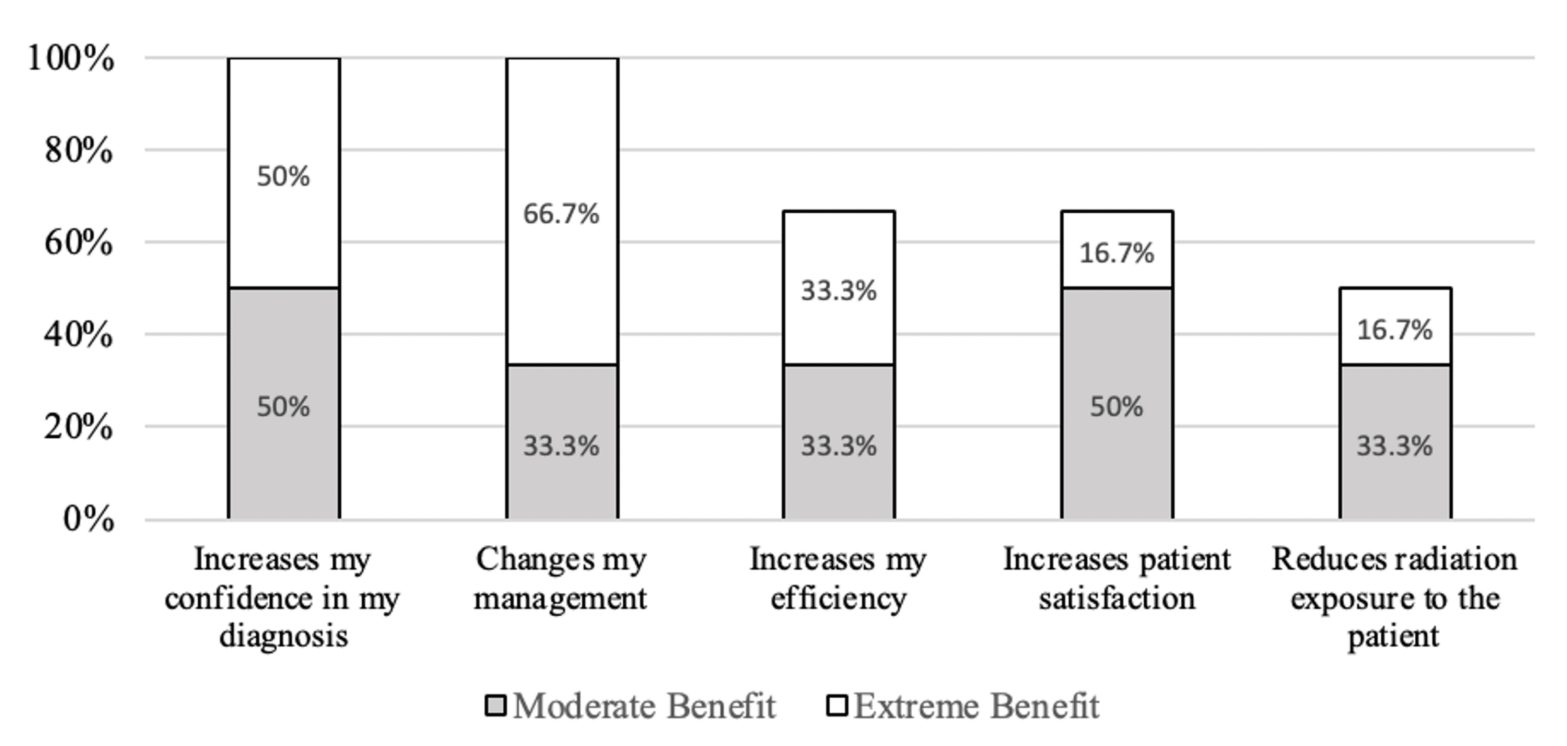
Percentage of APPs reporting a moderate or extreme benefit in response to the question: “Please rate the following benefits of performing POCUS: increases my confidence in my diagnosis, changes my management, increases my efficiency, increases patient satisfaction, and reduces radiation exposure to the patient.” Responses are based on a 4-point scale: 1 = not a benefit, 2 = somewhat of a benefit, 3 = moderate benefit, and 4 = extreme benefit APPs: advanced practice providers, POCUS: point-of-care ultrasound

The perceived benefits of POCUS were “increases my confidence in my diagnosis” (n=6, 100% moderate or extreme benefit), “changes my management” (n=6, 100% moderate or extreme benefit), “increases my efficiency” (n=4, 66.7% moderate or extreme benefit), “increases patient satisfaction” (n=4, 66.7% moderate or extreme benefit), and “reduces radiation exposure to the patient” (n=3, 50% moderate or extreme benefit) demonstrated in Figure 3.

**Figure 4:**
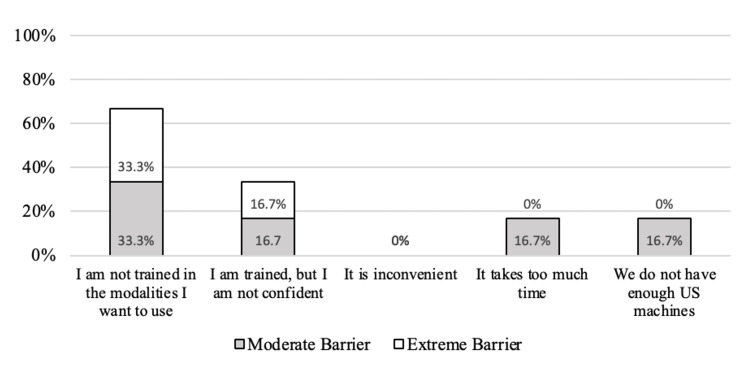
Percentage of APPs reporting a moderate or extreme barrier in response to the question: “Please rate the following barriers to performing POCUS: I am not trained in the modalities I want to use, I am trained but not confident, it is inconvenient, it takes too much time, and we do not have enough US machines.” Responses are based on a 4-point scale: 1 = not a barrier, 2 = somewhat of a barrier, 3 = moderate barrier, and 4 = extreme barrier APPs: advanced practice providers, POCUS: point-of-care ultrasound, US: ultrasound

The most commonly perceived barrier was “I am not trained in the modalities I want to use” when asked about training (n=4, 66.7% moderate or extreme barrier), confidence (n=2, 33.3% moderate or extreme barrier), time (n=1, 16.7% moderate or extreme barrier), availability of US machines (n=1, 16.7% moderate or extreme barrier), and inconvenience (n=0, 0% moderate or extreme barrier) in Figure 4.

**Figure 5:**
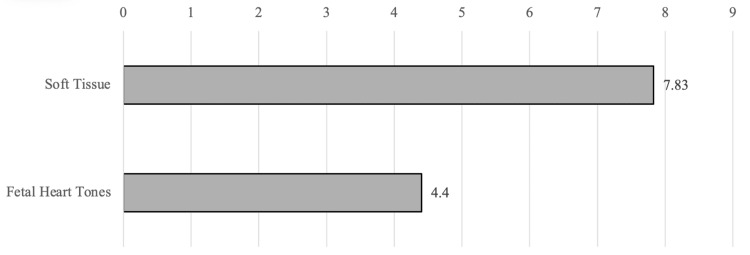
Mean self-reported confidence level of APPs in using POCUS for specific modalities, based on responses to the question: “My degree of confidence using POCUS (0 = least confident, 10 = most confident).” Reported confidence levels are provided for ST (N=6) and FHT (N=5) APPs: advanced practice providers, POCUS: point-of-care ultrasound, ST: soft tissue, FHT: fetal heart tone

The mean self-reported confidence (0 = least confident, 10 = most confident) in performing POCUS was 7.83 for ST (six responses) and 4.4 for FHT (five responses), as shown in Figure 5.

## Discussion

This study provides a comprehensive overview of four years of POCUS use by APPs in a large, academic, adult ED, focusing on the number and type of scans performed, QA of image acquisition and documentation, and insights from a voluntary survey of APPs.

Following the training protocol, an impressive 100% of APP-performed exams met the quality criteria for billable POCUS scans during the study period. The only noted barriers to billing were occasional lapses in documentation, which were promptly corrected through immediate feedback provided during routine chart completion communication.

POCUS has proven to be a valuable diagnostic tool in emergency settings, improving patient care and offering cost-effective alternatives to traditional imaging modalities [20]. However, data on the prevalence of POCUS use and billing in the ED remain limited [21]. In this study, the billable status of POCUS exams was used as a surrogate for quality and clinical appropriateness, as meeting billing criteria required high-quality imaging, accurate documentation, and adherence to clinical guidelines. This metric served as a practical measure in our QA process.

Notably, only two of the APPs who completed the survey responded "yes" to the question regarding billing for POCUS exams. This finding highlights an important opportunity to incorporate billing education into future didactic sessions. Moving forward, our curriculum will explicitly include institution-specific billing practices to ensure APPs are well-informed on this aspect of POCUS utilization.

Our study demonstrates that targeted training, combined with a system that allows for ongoing practice and competency achievement, can facilitate the integration of POCUS into the care of ED patients by APPs.

Additionally, APPs identified barriers to POCUS adoption, with a lack of training and access to certain modalities being the most prominent challenges. Addressing these barriers through structured education and improved resources may further enhance the incorporation of POCUS in clinical practice.

Survey responses highlighted the perceived value of POCUS among APPs. Respondents identified multiple benefits of POCUS in the ED and demonstrated enthusiasm for expanding their skills despite training being limited to two modalities (ST and FHT). The largest barriers to POCUS use were associated with limited training opportunities rather than challenges inherent to the technology itself. This finding underscores the potential value of expanding POCUS training for APPs to include additional applications and providing refresher courses to reinforce previously learned skills.

Interestingly, while APPs could perform billable FHT POCUS exams accurately, they reported lower confidence in this modality compared to ST POCUS. This may be attributed to the complexity of using M-mode for FHT or the emotional difficulty hesitancy associated with detecting the absence of fetal heartbeat in desired pregnancies.. These observations suggest a need for continued support and targeted training to build confidence in more complex applications of POCUS.

Prior research demonstrates the broad utility of POCUS in EM, with benefits including improved diagnostic accuracy, reduced time to consultation, shorter ED length of stay, and avoidance of ionizing radiation [22-24]. Furthermore, retrospective reviews have suggested that POCUS could prevent a significant proportion of morbidity and mortality in emergency cases, with the majority of POCUS exams providing clinical value [25]. These findings align with the favorable outcomes observed in this study, where APPs could perform high-quality, billable scans with appropriate training and support.

Limitations

This study has several limitations. It was conducted at a well-resourced, single academic institution with a relatively small cohort of APPs and focused primarily on ST and FHT POCUS. Despite email reminders, only about half of the APPs completed the voluntary survey, introducing non-responder bias and limiting the generalizability of the survey results.

Additionally, all POCUS examinations were reviewed by a single, board-certified, ultrasound fellowship-trained emergency physician with extensive experience, potentially introducing reviewer bias. The study was conducted in an academic ED with a well-established QA process and a supportive environment for POCUS training. Faculty members were experienced in teaching POCUS to learners, including residents and fellows, creating an ideal setting for training APPs. These factors may make replication challenging in resource-limited settings or institutions without established POCUS programs.

The APPs in this study were also experienced ED practitioners with a baseline familiarity with POCUS, which may not be representative of APPs in other settings, particularly those with less experience or exposure to ultrasound.

Future directions

Future research should explore the replicability of this training model at other institutions and examine the impact of expanding POCUS training for APPs to additional modalities, such as FAST and cardiac ultrasound. Investigating how POCUS impacts specific APP performance metrics, such as patients per hour, provider-to-decision time, length of stay, and work relative value units, would also be valuable.

Comparative studies evaluating ED length of stay for patients managed by APPs trained in POCUS versus those requiring formal ultrasound or relying on attending physicians for POCUS exams could provide further insights. Additionally, examining the efficiency of APPs performing POCUS independently versus deferring to attending physicians could clarify the optimal workflow, particularly in different ED care models, such as fast-track areas or collaborative staffing arrangements.

By addressing these questions, future research could further define best practices for integrating POCUS into APP workflows and enhance the overall quality of care provided in emergency settings.

## Conclusions

This study demonstrates that APPs can successfully incorporate ST and FHT POCUS into their clinical practice with focused training and ongoing QA. Using a structured training program, including a simulation-based session, direct supervision of 10 scans, and continuous quality review, APPs at a large, academic, adult ED were able to perform high-quality, billable POCUS exams.

APPs identified multiple benefits of POCUS, highlighting its value as a diagnostic and procedural tool in EM. However, they also noted that limited training opportunities in additional modalities remain a significant barrier to broader POCUS utilization. 

## Appendices

**Table 2:** Median APP responses to the question, “How often do you personally use the POCUS application?” Responses are based on a 5-point scale: 1 = never, 2 = rarely, 3 = sometimes, 4 = often, and 5 = very often APP: advanced practice provider, POCUS: point-of-care ultrasound

POCUS application		ST	Cardiac	FHT	Vascular access	Other
N		6	6	6	6	5
Median		4.00	1.00	2.00	1.00	1.00
Percentiles	25	3.00	1.00	1.00	1.00	1.00
	75	4.25	1.75	3.25	2.25	2.50

**Table 3:** Median APP responses to the prompt: "Please rate the following benefits of performing POCUS: increases my confidence in my diagnosis, changes my management, increases my efficiency, increases patient satisfaction, and reduces radiation exposure to the patient.” Responses are based on a 4-point scale: 1 = not a benefit, 2 = somewhat of a benefit, 3 = moderate benefit, and 4 = extreme benefit APP: advanced practice providers, POCUS: point-of-care ultrasound

Confidence		Increases my confidence in my diagnosis	Changes in my management	Increases my efficiency	Increases patient satisfaction	Reduces radiation exposure to the patient
N		6	6	6	6	6
Median		3.50	4.00	3.00	3.00	2.50
Percentiles	25	3.00	3.00	1.75	2.00	1.00
	75	4.00	4.00	4.00	3.25	3.25

**Table 4:** Median APP responses to the prompt: "Please rate the following barriers to performing POCUS: I am not trained in the modalities I want to use, I am trained but not confident, it is inconvenient, it takes too much time, and we do not have enough US machines.” Responses are based on a 4-point scale: 1 = not a barrier, 2 = somewhat of a barrier, 3 = moderate barrier, and 4 = extreme barrier APP: advanced practice providers, POCUS: point-of-care ultrasound, US: ultrasound

Barrier		I am not trained in the modalities I want to use	I am trained, but I am not confident	It is inconvenient	It takes too much time	We do not have enough US machines
N		6	6	6	6	5
Median		3.00	2.00	1.50	2.00	1.00
Percentiles	25	1.75	1.75	1.00	1.75	1.00
	75	4.00	3.25	2.00	2.25	2.50

**Table 5:** Mean self-reported confidence level of APPs in using POCUS for specific modalities, based on responses to the question: “My degree of confidence using POCUS (0 = least confident, 10 = most confident).” Reported confidence levels are provided for ST (N=6) and FHT (N=5) APPs: advanced practice providers, POCUS: point-of-care ultrasound, ST: soft tissue, FHT: fetal heart tone

Application		ST	Cardiac	FHT	Vascular access
N		6	2	5	3
Mean		7.83	3.50	4.40	3.33
SD		0.983	3.536	2.966	3.215
Percentiles	25	7.00	1.00	2.00	1.00
	75	9.00		7.50	
